# Supplementary material for: Dissipative Landau-Zener tunneling in the crossover regime from weak to strong environment coupling
Source: Nat Commun. 2025 Jan 2;16:329. doi: 10.1038/s41467-024-55588-x (PMC11697257; doi:10.1038/s41467-024-55588-x)
Supplement: Supplementary file 1 — Supplementary Information [file 41467_2024_55588_MOESM1_ESM.pdf]

Supplementary Information

CONTENTS

Supplementary Note 1. Wiring 1

Supplementary Note 2. Crosstalk Calibration 1

Supplementary Note 3. Circuit Model 2

Supplementary Note 4. Fast flux line coupling characterization 3

Supplementary Note 5. Fast flux line pulse distortion characterization 3

Supplementary Note 6. State preparation and readout calibration 5

Supplementary Note 7. Noise parameters and master equation simulation 7
A. Effect of X-noise coupling 8
B. Adiabatic master equation (AME) 8
C. Polaron-transformed master equation (PTRE) 9
D. Symmetric versus Asymmetric Landau-Zener Sweep 10

Supplementary Note 8. Anomalous Population inversion at $\Phi_x = 0.2$ 10

Supplementary Note 9. Spin bath 11
A. Single spin bath 11
B. Multiple spins 14

References 16

Supplementary Note 1. WIRING

In Supplementary Figure 1 we present a diagram of the room-temperature and cryogenic setup used in the experiments. The qubit flux biases for the x and z loops, Φ_x and Φ_z, are controlled by currents generated by a DC voltage source and a high bandwidth source, combined through a bias tee at the mixing chamber stage of the dilution refrigerator. The DC biases are supplied by Yokogawa GS 200 voltage sources through twisted pair cables and low-pass filtered to below 32 kHz to minimize noise. The high bandwidth components of the current are supplied by a 1 GHz arbitrary waveform generators (AWGs) (Keysight M3202A), through coaxial cables. The fast line coupling to Φ_x suffered from a cold open during the cooldown and is not used for the Landau-Zener experiment. The attenuators and filters on the cable are chosen to allow a sufficiently large current range for driving Rabi oscillation and performing annealing experiment, while minimizing decoherence due to thermal and electronic noise. The resonator SQUID bias is supplied by an AWG (Keysight M3202A), but through twisted pair cable. The qubit can be driven with a capacitively coupled driving line, supplied by an rf source with an integrated IQ modulator, through a coaxial cable. The readout signal is amplified by a travelling wave parametric amplifier (TWPA) [S1] at the mixing chamber stage of the fridge, followed by a high-electron mobility transistor amplifier (HEMT) at the 4K stage, and room temperature amplifiers, before being processed by a field-programmable gate array (FPGA) digitizer (Keysight M3102A).

Supplementary Note 2. CROSSTALK CALIBRATION

DC flux crosstalk between different bias lines and flux loops are calibrated using the CISCIQi method developed in Ref. [S2]. We first measure the flux bias dependent resonator spectrum and the crosstalk into the resonator from other bias lines. This allows us to fix the resonator bias to measure the qubit-bias dependent transmission through the resonator. The procedure is iterated a few times until the crosstalk is compensated to within $1\,\mathrm{m}\Phi_0$ accuracy. The full crosstalk matrix is shown in Supplementary Figure 2(a).

Crosstalk from the fast pulses sent to the qubit z loop to other loops are not compensated due to bandwidth limitations on other bias lines. For the small pulse amplitude used for the Landau-Zener sweep, reaching up to $10\,\mathrm{m}\Phi_0$, the induced flux on the x, r loop should be inconsequential.

Supplementary Note 3. CIRCUIT MODEL

The device used in this experiment consists of a capacitively-shunted flux qubit coupled to a tunable rf-SQUID terminated resonator. The coupling between these two circuits is done via the mutual inductance between the qubit z loop and the rf-SQUID loop. A lumped element representation of the qubit and resonator circuit is shown in Supplementary Figure 2(b). The qubit eigenstates and eigenvalues are obtained using the numerical tools developed in Ref. [S3]. The circuit persistent current I_p and gap Δ were verified experimentally via qubit spectroscopy, as shown in Supplementary Figure 2(c). The simulated I_p and Δ values are plotted as a function of Φ_x in Supplementary Figure 2(d).

The parameters of the resonator rf-SQUID were determined from fitting the experimental values of the resonator

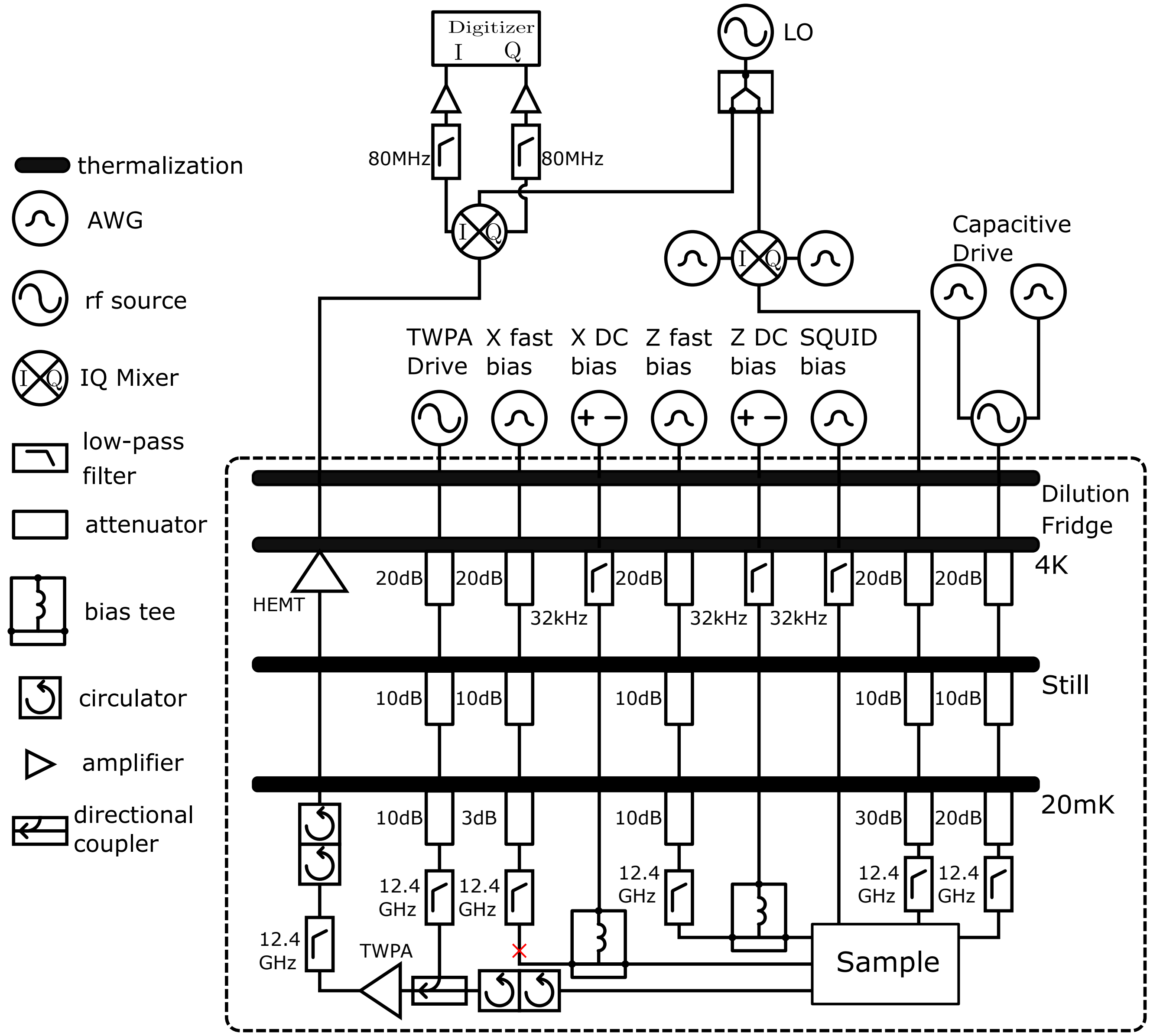

Supplementary Fig. 1. Schematic of the setup used for experiments. The open fast X-bias line is marked in red cross.

L_r	16.4pH
L_l	16.4pH
L_z	799.8pH
$I_{\rm cr}$	77.6nA
$I_{\rm cl}$	89.1nA
$I_{\rm c1}$	244.7nA
$I_{\rm c2}$	244.7nA
$C_{\rm cd}$	0.4fF
$C_{\rm ad}$	3.6fF
$C_{\rm ac}$	0.04fF
$C_{\rm ab}$	14.2fF
$C_{\rm gd}$	44.1fF
$C_{\rm gc}$	0.2fF
$C_{\rm ga}$	141.4fF
$C_{\rm gb}$	90.0fF
SQUID inductance L_s	238.7pH
SQUID junction I_s	1187nA
resonator length l_r	3.364mm
Mutual between qubit and SQUID M	60.3pH

Supplementary Tab. I. Fit model best parameters for the qubit and resonator circuits.

frequency versus resonator flux bias Φ_r, shown in Supplementary Figure 3(a). The resonator model allows for extraction of the value of the screening current in the rf-SQUID, which was used to determine the shift in the symmetry point of the qubit, induced by the rf-SQUID when biased away from zero. In order to confirm this value experimentally, spectroscopy curves were taken at a fixed value of Φ_x at two different values of resonator bias Φ_r at zero and -0.15, the later being the value used for persistent current readout. The two spectroscopy curves are shown in Supplementary Figure 3(b). The circuit parameters are summarized in Supplementary Table I.

Supplementary Note 4. FAST FLUX LINE COUPLING CHARACTERIZATION

As described in Supplementary Note Supplementary Note 1, fast voltage pulses are applied to the qubit z loop via a bias tee in order to control the flux bias of the qubit during the Landau-Zener sweep. In order to determine the transfer function between the voltage of the AWG and the flux fed to the qubit loop, a procedure using Ramsey interferometry was used.

The protocol is shown in Supplementary Figure 4(a). Two $\pi/2$ pulses are applied using the capacitively coupled waveguide, separated by the time $\tau_{\rm delay}$. During the interval between the two $\pi/2$ pulses, a trapezoidal flux pulse with 1 ns rise and fall time is applied to the z loop of the qubit. This flux pulse adiabatically changes the qubit frequency, depending on the pulse amplitude and duration, which induces an additional phase for the superposition created by the first $\pi/2$ pulse, inducing an oscillation whose period depends on the flux amplitude. An example is shown in Supplementary Figure 4(b) for the case with no flux pulse applied (i.e. pure Ramsey with no detuning) and with a 60 mV pulse applied.

The above sequence is repeated at a range of pulse amplitudes such that the detuning varied between approximately from 0 to 60 MHz. This detuning versus flux pulse amplitude is shown in Supplementary Figure 4(c). Combining the detuning and the drive frequency gives the effective qubit frequency during the delay time in the Ramsey sequence. Comparing the qubit frequency versus flux pulse amplitudes allows us to deduce the voltage to flux conversion for the fast flux line.

Supplementary Note 5. FAST FLUX LINE PULSE DISTORTION CHARACTERIZATION

During the Landau-Zener measurement sequence, time-dependent flux pulses are applied to the qubit via a bias tee, as discussed in Supplementary Note 1 and Supplementary Note 4. As the experiment involved the application of long pulses (duration $> 1\ \mu$s), we characterize the transmission of the AC port of the bias tee to check for possible frequency dependent attenuation effects that would distort the pulse shape.

In order to check for distortion effects, we used an experimental protocol based on a Ramsey sequence, shown in Supplementary Figure 5(a). Two microwave $\pi/2$ pulses are applied with a fixed delay time $\tau_{\rm delay}$. The delay

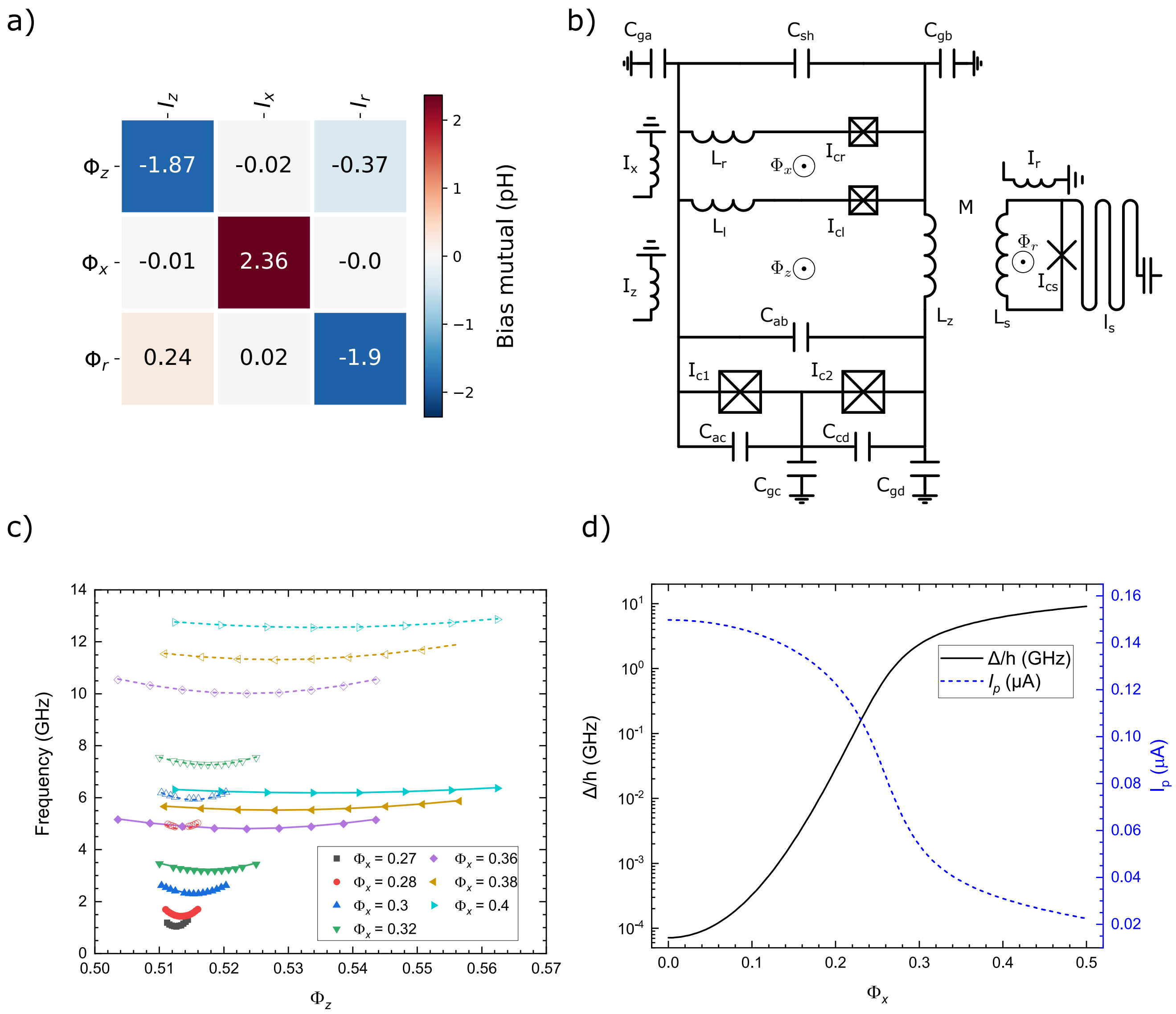

Supplementary Fig. 2. a) Matrix of mutual inductances between on chip bias lines and flux loops.. b) Circuit diagram for the qubit and coupler. c) Experimentally measured qubit transition frequency and simulated qubit transition frequencies for the best fit parameters, as a function of the biases Φ_x and Φ_z, and comparison with experiment. Filled symbols (solid lines) correspond to the experimentally obtained (simulated) transition frequencies between the ground and first excited state. Open symbols (dashed lines) correspond to the experimental (simulated) transition frequencies between the ground and second excited state. d) Simulated minimum gap (left axis, solid curve) and persistent current (right axis, dashed curve) values as a function of the bias Φ_x.

time is chosen to correspond to the inflection point of one of the Ramsey oscillations, so that the readout signal is maximally sensitive, and responds linearly to changes in qubit frequency and hence the flux pulse amplitude. The spacing between the Ramsey sequence and readout is fixed. In addition, a square pulse from Φ_z fast line is applied with duration $\tau_{\rm flux}$. The position of the rising edge of the square pulse is varied, spanning a range of times relative to the Ramsey pulses, from the rising edge following the first $\pi/2$ pulse to preceding the first $\pi/2$ pulse. This is done by increasing $\tau_{\rm flux}$ while keeping the falling edge and the Ramsey pulses at a fixed position relative to the readout pulse.

The readout signal as a function of pulse duration is shown in Supplementary Figure 5(b) for several values of pulse amplitudes. For short pulse duration, the pulse starts after the Ramsey sequence and does not change the readout signal. As the pulse duration increases, the leading edge of the pulse moves past the second and then the first $\pi/2$ pulse. The flux pulse experienced by the qubit in between the two $\pi/2$ pulses changes the phase of the Ramsey oscillation, causing a sharp change in the measured signal, with width corresponding to $\tau_{\rm delay}$. The Ramsey signal shown in Supplementary Figure 5(b) is flat up to flux pulse duration $\tau_{\rm flux}$ as long as 30 μs, indicating that the pulse

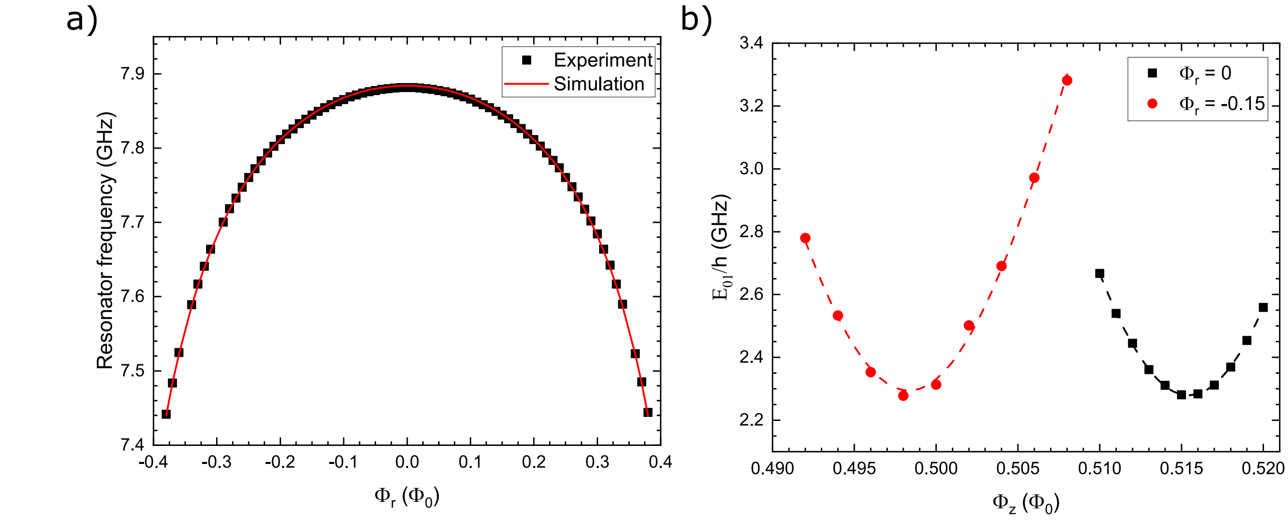

Supplementary Fig. 3. a) Readout resonator resonance frequency vs bias: experiment (solid points) and simulation with best fit parameters (solid line). b) Experimentally measured shift of the qubit z symmetry point due to the SQUID screening current. Solid symbols correspond to qubit frequencies determined from spectroscopy, and dashed lines correspond to a fit using a two-level system anticrossing relation.

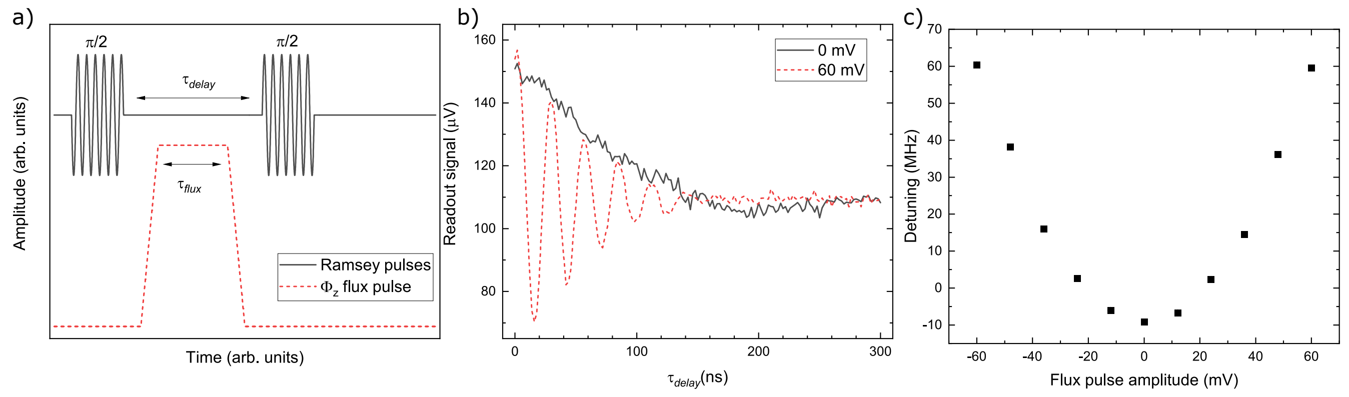

Supplementary Fig. 4. Bias line characterization using a Ramsey protocol. a) Pulse sequence for Ramsey measurements. The qubit is initialized in a superposition using a $\pi/2$ pulse, then a flux pulse applied to the qubit Z bias adiabatically detunes the qubit away from the symmetry point. The spacing between the two $\pi/2$ pulses $\tau_{\rm delay}$ equal to the pulse duration $\tau_{\rm flux}$ plus the rise and fall times. The qubit acquires a phase dependent on the amplitude and duration of the pulse. b) Ramsey oscillation curves with $V_z = 0$ and 60 mV. c) Fitted detuning as a function of pulse amplitude.

is negligibly distorted at these time scales.

Supplementary Note 6. STATE PREPARATION AND READOUT CALIBRATION

In this section, we describe the protocol for preparing the qubit in its ground state and the method for calibrating the readout voltage to obtain the state populations.

The qubit can in general be prepared in the ground state by waiting long enough. However, for some of the small gap Δ values used in the Landau- Zener experiments, the transition rate between the ground states of the two wells is very slow. Therefore, to prepare the qubit in the ground state before the Landau-Zener measurements, we use a sideband cooling method similar to that used in Ref. [S4] A plot of the qubit energy levels is shown in Supplementary Figure 6. The qubit is prepared in its ground state at a bias about $\Phi_z - \Phi_{z,\rm sym} \approx 0.005$ away from the symmetry point, as follows. A sinusoidal pulse is applied which, on its positive side, sweeps the qubit further away from the symmetry point and past the anti-crossing between the first and second excited states. Prior to this sinusoidal pulse, the qubit is in a mixture of the ground and excited states. Due to the larger anti-crossing between the higher levels,

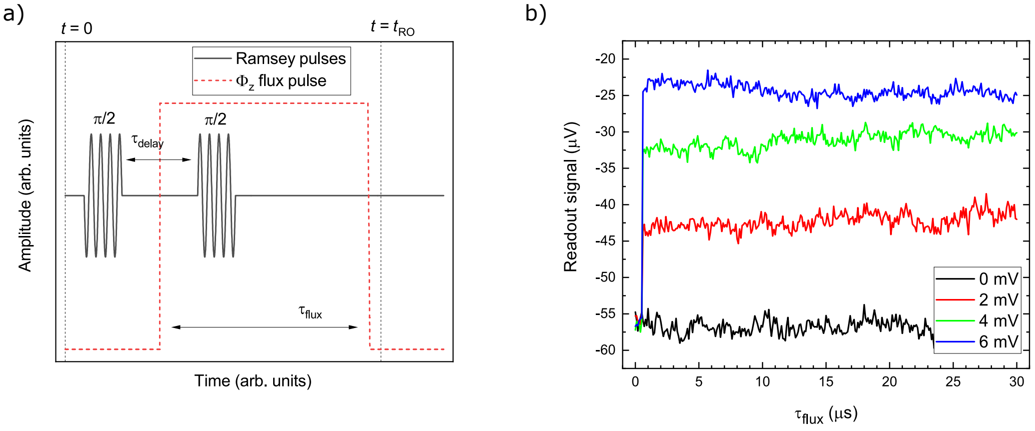

Supplementary Fig. 5. Characterization of pulse distortion from the bias tee. a) Schematic of Ramsey-based sequence used to quantify the pulse distortion. b) Readout voltage as a function of pulse duration τ_{flux}. The flat profile of the readout voltages out to several tens of μs shows that the bias tee negligibly distorts the pulse shape.

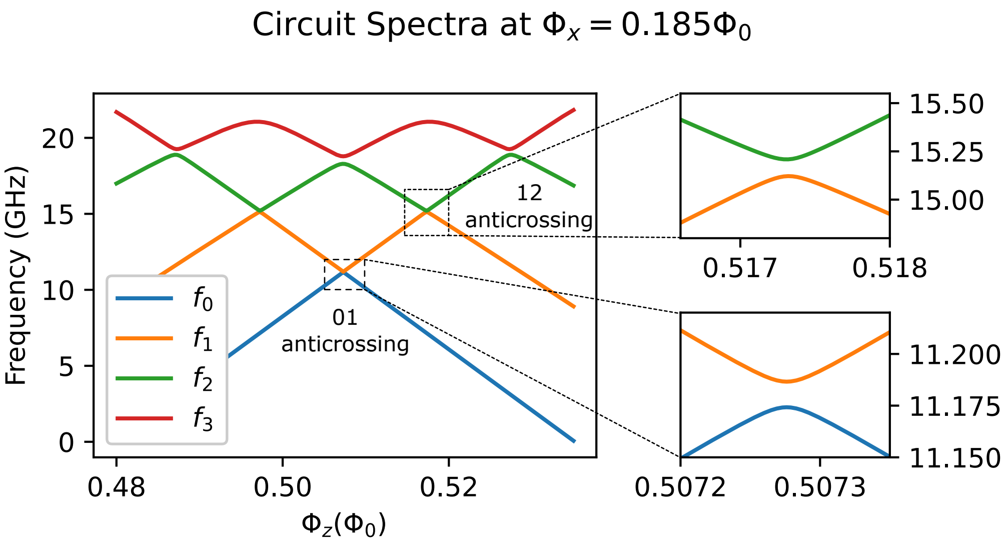

Supplementary Fig. 6. Energy Spectrum of the qubit circuit at $\Phi_x = 0.185\Phi_0$, with close up of the 01 and 12 anticrossing

the excited state can be adiabatically transferred into the same well as the ground state, which allows fast relaxation back to the ground state. By repeated sweeping across the anti-crossing between the first and second excited state, it is possible to prepare the qubit in the ground state. In our experiments, the qubit was cycled 5-10 times in order to prepare the ground state prior to the Landau-Zener sweep.

Next, we discuss the calibration of the readout signal. Given a fixed readout frequency, the transmission measured is given by a complex number $V_g (V_e)$ for the qubit at the ground (excited) state. For a qubit in the mixed state, the readout voltage is given by $V = V_g + P_e(V_e - V_g)$ where we assume populations beyond the qubit states are negligible. Therefore to obtain the qubit excited state population we need to obtain V_g and V_e.

For the Landau-Zener measurements, V_g and V_e are calibrated at each Φ_x. To measure V_g we prepare the qubit in the ground state at the readout point (at the end of the Landau-Zener sweep) using the cooling procedure discussed above. To measure V_e, we prepare the qubit in the excited state by preparation in the ground state at the opposite side of the symmetry point, followed by a fast, 1 ns long, Landau-Zener sweep through the minimum gap. Taking the qubit model parameters and the coherent Landau-Zener formula, a 1 ns Landau-Zener is expected to lead to a final excited state probability larger than 99% for even the largest Δ measured in our experiments.

Supplementary Note 7. NOISE PARAMETERS AND MASTER EQUATION SIMULATION

In this section, we discuss the noise model used in the master equation simulation. We first introduce the general form of noise and then discuss some specificities regarding including them into the adiabatic master equation (AME) and polaron-transformed Redfield equation (PTRE). For reference, we also provide a comparison of the noise parameters used in this work with noises measured in three other flux-qubit based quantum annealing devices, as summarized in Supplementary Table II.

As the qubit circuit has relatively large flux loops, we assume the flux noise in the qubit x and z loops are the dominant sources of noise. The noises lead to fluctuation in the circuit Hamiltonian via

$$\delta H_{\mathrm{c}}(\Phi_z, \Phi_x) = \sum_{\lambda \in \{\Phi_z, \Phi_x\}} \frac{\partial H_{\mathrm{c}}}{\partial \lambda} \delta\lambda. \tag{S1}$$

In particular for $\lambda = \Phi_z$, in the two level approximation $\frac{\partial H_{\mathrm{c}}}{\partial \Phi_z} = -I_p \sigma_z$. Note here when the noise source is quantum, $\delta\lambda$ is a quantum operator of the environment.

The noise power spectral density (PSD) due to $\delta\lambda$ is given by the Fourier transform of its auto-correlation function,

$$S_\lambda(\omega) = \int_{-\infty}^{\infty} \mathrm{d}\tau e^{i\omega\tau} \langle \delta\lambda(\tau)\delta\lambda(0)\rangle. \tag{S2}$$

Various previous measurements have shown that flux noise has roughly $1/f$ dependence, with f being frequency, up to around 1GHz, and then a quasi-ohmic spectrum at higher frequencies [S5–S7]. Furthermore, we follow Ref. [S6] to consider a quantum noise PSD with the positive and negative frequency components related by a phenomenological thermodynamic model. These considerations lead to the noise PSD given by

$$S_\lambda = S_{\lambda,1/\mathrm{f}} + S_{\lambda,\mathrm{ohmic}} \tag{S3}$$

$$S_{\lambda,1/\mathrm{f}} = \frac{A_\lambda \omega}{|\omega|^\alpha}\left[1 + \coth\left(\frac{\beta\hbar\omega}{2}\right)\right], \tag{S4}$$

$$S_{\lambda,\mathrm{ohmic}} = B_\lambda \omega |\omega|^{\gamma-1}\left[1 + \coth\left(\frac{\beta\hbar\omega}{2}\right)\right], \tag{S5}$$

where $\beta = 1/k_B T$ is the inverse temperature, A_λ, B_λ determines the noise strength and α, γ determine the frequency dependence. For $1/f$ noise, $\alpha = 1$ and for ohmic noise $\gamma = 1$. The temperature is assumed to be close to the base temperature of the fridge, $T = 20\,\mathrm{mK}$.

The parameters of $1/f$ noise are obtained via measuring the flux-bias dependent Ramsey dephasing times (see Supplementary Figure 7). The Ramsey dephasing time probes symmetrized $1/f$ noise in the low-frequency limit, satisfying $\hbar\omega \ll k_B T$. In this limit, we have

$$S^+_{\lambda,1/f}(\omega) = \frac{1}{2}\left(S_{\lambda,1/f}(\omega) + S_{\lambda,1/f}(-\omega)\right) \tag{S6}$$

$$\approx \frac{A_\lambda \omega}{|\omega|^\alpha} \frac{2}{\hbar\omega\beta} \tag{S7}$$

$$= A^*_\lambda \left(\frac{2\pi}{|\omega|}\right)^\alpha, \tag{S8}$$

where we defined $A^*_\lambda = 2A_\lambda/[\hbar\beta(2\pi)^\alpha]$ to relate to the more commonly used expression for $1/f$ flux noise, used in for example Ref. [S8]. Given the similarity between our device and the device used in Ref. [S8], we assume $\alpha = 0.91$ and found $A^*_{\Phi_z} = \left(8.7 \times 10^{-6}\right)^2 \Phi_0^2/\mathrm{Hz}$, $A^*_{\Phi_x} = \left(5. \times 10^{-6}\right)^2 \Phi_0^2/\mathrm{Hz}$ fits the measured Ramsey dephasing time best. More details on the coherence characterization of this qubit are discussed in a separate publication [S9]. The quasi-ohmic component of the flux noise mainly contributes to qubit relaxation. It is more difficult to give a quantitative estimate of the quasi-ohmic noise power as it leads to similar flux bias dependence of relaxation rates as other noise sources, such as ohmic charge noise [S5, S6]. For this reason, we use the reported ohmic noise strength measured in Ref. [S6] and scale it according to the ratio of the $1/f$ noise strength at 1Hz between the two devices. This gives $\gamma = 1$ and $B_{\Phi_z} \approx 2.7 \times 10^{-30}\Phi_0^2/\mathrm{Hz}^2, B_{\Phi_x} \approx 9.2 \times 10^{-31}\Phi_0^2/\mathrm{Hz}^2$. These values give reasonable agreement with the qubit T_1 relaxation times we have measured. In Supplementary Table II, we also provide these numbers in terms of the dimensionless coupling constant η that is often discussed in spin-boson literature. We also want to note that the simulation result is largely unchanged if the ohmic component of the noise spectrum is not included.

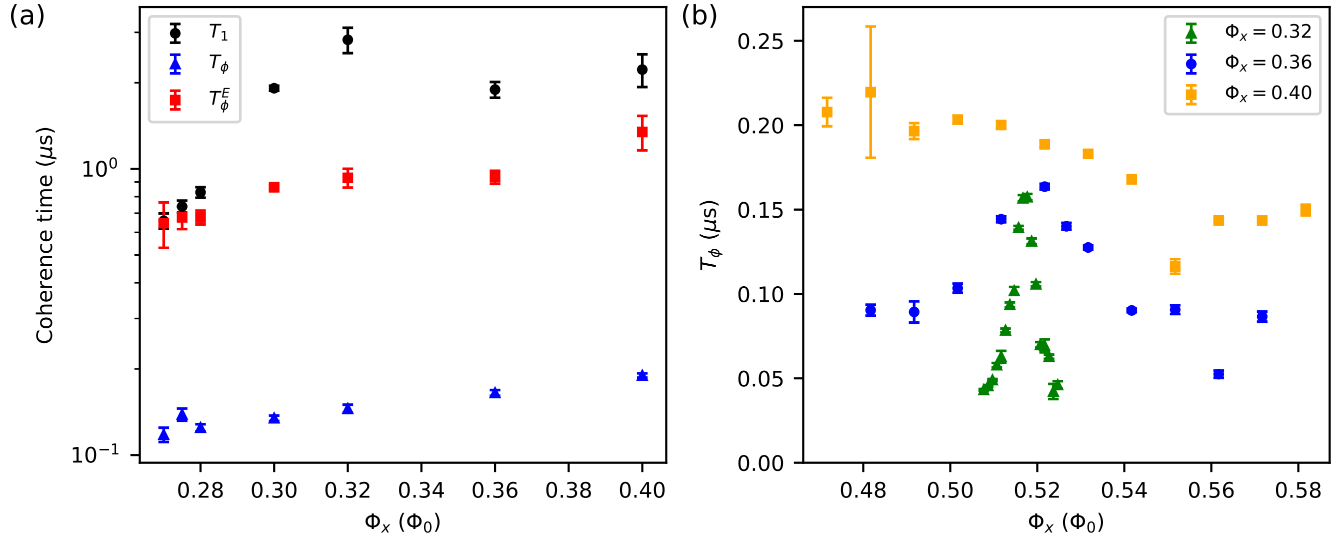

Supplementary Fig. 7. Measured coherence times of the device. a) The measured relaxation time (T_1), Ramsey and spin-echo pure dephasing time (T_ϕ, T_ϕ^E) at the symmetry points of Φ_z for different Φ_x. b) The measured Ramsey pure dephasing time T_ϕ at different Φ_z for three different Φ_x values. The measured coherence times are used to find the noise parameters used in the master equation simulations.

	This work	Quintana et. al [a]	DWave CJJ qubit [b]	DWave CCJJ qubit [c]
$A^*_{\Phi_z}$ (Φ_0^2/Hz)	$(8.7 \times 10^{-6})^2$	$\sim (5 \times 10^{-6})^2/2$		$(1.3 \times 10^{-6})^2/2$
α	0.91	$0.96 - 1.05$		0.95
$I_p(\mu\text{A})$	$0.104 - 0.129$	~ 0.5		~ 1.0
W/h(predicted)(GHz)	$0.048 - 0.059$	~ 0.16		0.05
W/h(measured)(GHz)		~ 0.25	2.6	1.4
T_{eff}(mK)[d]	20	20	21	53
ηg^2[e]	1.4×10^{-5}	$\sim 5 \times 10^{-5}$		~ 0.065[S7]

[a] The numbers are based on Ref. [S6]
[b] The numbers are based on Ref. [S10] where the Landau-Zener experiment is performed.
[c] The numbers are based on Ref. [S7, S11]
[d] This is the effective temperature that describes the MRT data assuming low-frequency noise is at thermal equilibrium.
[e] ηg^2 is defined such that, the system bath coupling is given as $gA \otimes B$, where A, B are norm-1 system and bath operators respectively and the noise PSD of B is given as $S(\omega) = 2\pi\eta\hbar^2\omega\frac{\exp(-|\omega|/\omega_c)}{1-\exp(-\beta\hbar\omega)}$. See for example Ref. [S12] for more details.

Supplementary Tab. II. Comparison of noise parameters used for simulation in this work and other work using flux qubits for quantum annealing.

A. Effect of X-noise coupling

In the master equation simulations, noise from Φ_x is not included. This is justified based on three considerations. Firstly, the noise power of Φ_x is less than half the noise power in Φ_z. Secondly, as plotted in Supplementary Figure 8, the matrix elements of the flux operators between the circuit energy eigenstates $\langle\alpha|\partial H/\partial\Phi_x|\beta\rangle$ is smaller than $\langle\alpha|\partial H/\partial\Phi_z|\beta\rangle$ by a factor of 10. Finally, Φ_x noise only primarily leads to transverse noise assuming small Φ_x and small x loop junction asymmetry. Previous studies suggested that for transverse noise to have similar dissipative effects, its coupling strength needs to be at least about 1/10 of the longitudinal noise [S13]; this condition is far from being satisfied in our case.

B. Adiabatic master equation (AME)

In order to implement in AME the noise PSD in Supplementary Eq. (S3), additional low- and high-frequency cutoffs ω_l and ω_h are added to avoid divergence. This introduces $S^{\text{AME}}_{\Phi_z}$, the PSD used in AME simulations, defined as

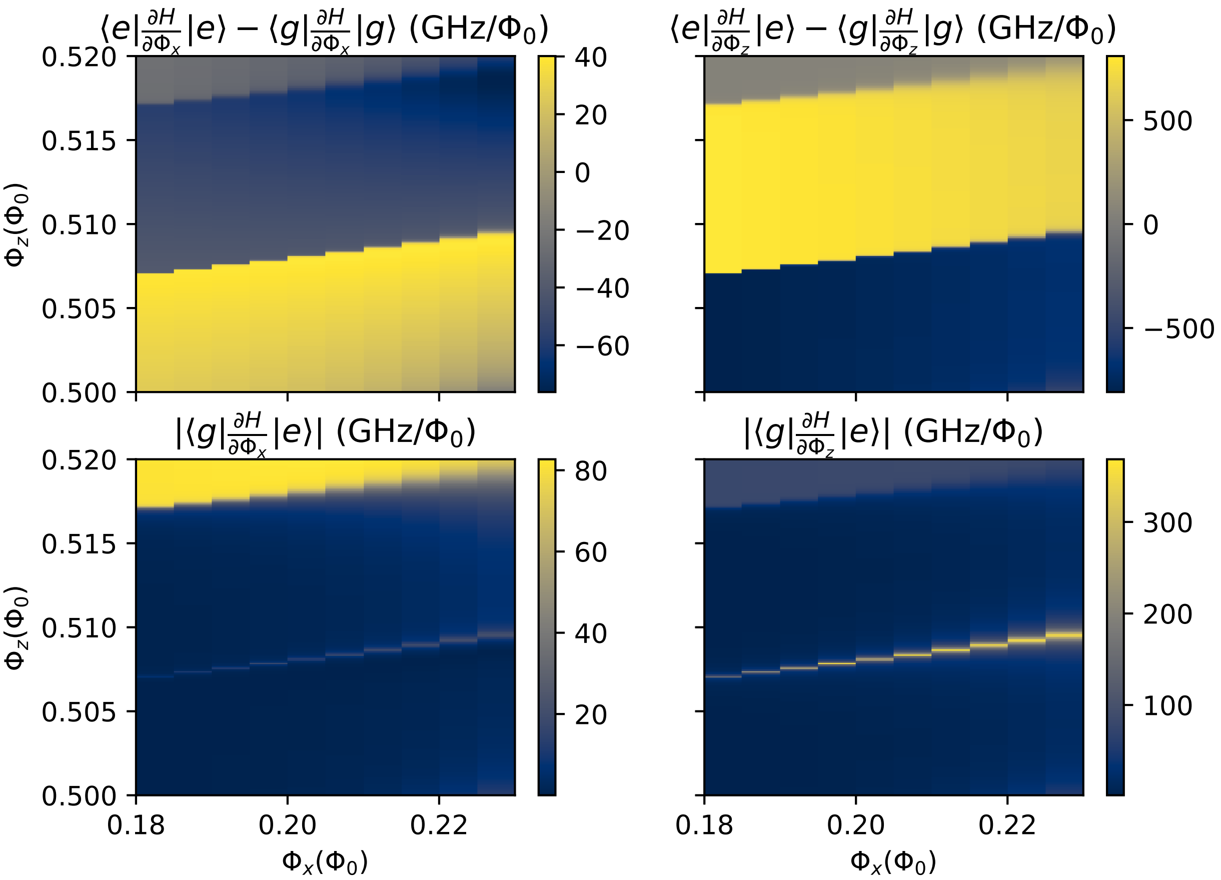

Supplementary Fig. 8. Matrix elements for the x and z flux bias.

$$S^{\mathrm{AME}}_{\Phi_z} = \begin{cases} S_{\Phi_z,1/\mathrm{f}}(\omega)\exp\left(\frac{-|\omega|}{\omega_h}\right) + S_{\Phi_z,\mathrm{ohmic}}(\omega)\exp\left(\frac{-|\omega|}{\omega_h}\right) & |\omega| > \omega_l \\ S_{\Phi_z,1/\mathrm{f}}(\omega_l)\exp\left(\frac{-|\omega_l|}{\omega_h}\right) + S_{\Phi_z,\mathrm{ohmic}}(\omega)\exp\left(\frac{-|\omega|}{\omega_h}\right) & |\omega| \leq \omega_l. \end{cases} \tag{S9}$$

The high-frequency cutoff is chosen to be $\omega_h/2\pi = 10$GHz, which is roughly the characteristic oscillation frequency in either of the qubit potential wells. For the low-frequency cutoff, given that we are primarily concerned with thermalization effects, we choose $\omega_l/2\pi$= 10MHz, which corresponds to the minimum qubit frequency for the Landau-Zener measurement presented in this work.

C. Polaron-transformed master equation (PTRE)

In the PTRE simulation, the noise model consists of an ohmic noise just as in AME, and the $1/f$ noise is represented by the MRT parameters W and ϵ_p [S14]. The MRT width W characterizes the integrated effect of the symmetrized low-frequency noise,

$$W^2 = 2I_p^2 \int_{\omega_{\mathrm{low}}}^{\omega_{\mathrm{high}}} \frac{d\omega}{2\pi} S^{+}_{\Phi_z,1/\mathrm{f}}(\omega). \tag{S10}$$

The anti-symmetrized low frequency noise $S^{-}_{\Phi_z,1/\mathrm{f}}(\omega) = 1/2(S_{\Phi_z,1/\mathrm{f}}(\omega) - S_{\Phi_z,1/\mathrm{f}}(-\omega))$ gives the reorganization

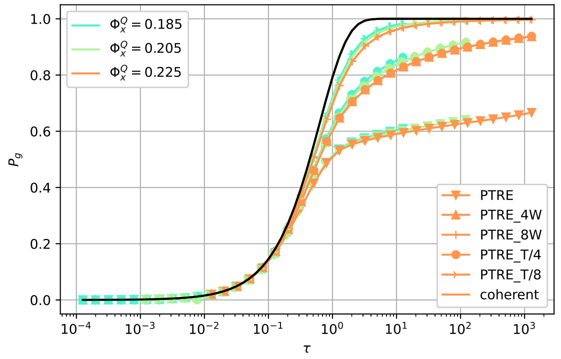

Supplementary Fig. 9. Final ground state probabilities versus the dimensionless sweep time $\tau = \Delta^2/\hbar v$ for different PTRE parameters.

energy ϵ_p

$$\epsilon_p = 2I_p^2 \int_{\omega_{\text{low}}}^{\omega_{\text{high}}} \frac{d\omega}{2\pi} \frac{S^{-}_{\Phi_z,1/\text{f}}(\omega)}{\hbar\omega}. \tag{S11}$$

For the integration limit, we choose $\omega_{\text{low}}/2\pi = 4\,\text{Hz}$ based on the experiment time taken for all the repetitions at each Φ_x and T_{LZ}, and $\omega_{\text{high}}/2\pi = 10\,\text{Ghz}$ based on the characteristic oscillation frequency in the qubit potential wells. We assume that the low-frequency noise is in thermal equilibrium, which relates W and ϵ_p via the fluctuation-dissipation theorem, $W^2 = 2k_B T \epsilon_p$. We also note that $1/f$ noise has a significant contribution to the noise power at high frequency, up to around 1 GHz. This contribution breaks the normalization condition for the high-frequency noise in the current numerical implementation of PTRE (see discussion around Eq. [16] of Ref. [S15]). The effect of the high-frequency component of $1/f$ noise in the strong coupling limit is to be explored in future work.

As discussed in the main text, the values of W or T need to be adjusted for better agreement between PTRE simulation and the experiment data at low Φ_x. The results are shown in Supplementary Figure 9. Interestingly, it is found that increasing W by 4(8) times is equivalent to decreasing T by 4(8) times. This indicates that the ratio ϵ/W is most critical to the result, with increasing ϵ_p/W leading to closer to the coherent limit of ground state population.

D. Symmetric versus Asymmetric Landau-Zener Sweep

As discussed in Supplementary Note 3, the screening current in the rf-SQUID leads to an effective bias to the qubit z loop. Due to an initial inaccurate estimation of this effect, the Landau-Zener data presented in the main text has an asymmetric scan range, with initial and final z loop bias being $\Phi_{z,\text{init}} = -3.1 \times 10^{-3}\Phi_0$ and $\Phi_{z,\text{final}} = 6.9 \times 10^{-3}\Phi_0$. This was later identified via the spectroscopy method mentioned in Supplementary Note 3, but leaving insufficient time to repeat the full range of Landau-Zener experiments. However, with this asymmetric scan range, the validity of the Landau-Zener model is not affected, since the initial and final longitudinal field are still much larger than the tunneling amplitude. In Supplementary Figure 10, we compare the measured and simulated results for symmetric and asymmetric Φ_z sweep range. The experiment data show some differences but the qualitative features discussed in the main text remain the same. In particular the symmetric data also show that as Φ_x decreases, P_g becomes closer to the coherent limit behaviour. The simulated results using either AME or PTRE for the symmetric and asymmetric sweep range do not differ significantly.

Supplementary Note 8. ANOMALOUS POPULATION INVERSION AT $\Phi_x = 0.2$

As noted in the main text, in the Landau-Zener measurement, the final ground state probability, P_g for $\Phi_x = 0.2$, drops below 0.5 for intermediate sweep time. This is inconsistent with the interpretation that the dominant mechanism determining P_g at intermediate sweep time is thermalization that occurs near the minimum gap. A possible explanation is imperfect ground state preparation just before the Landau-Zener sweep, which affects both the Landau-Zener measurement itself and the calibrated V_e. Assuming the ground state initialization procedure leaves a finite population in the initial excited state, the result of the Landau-Zener measurement is shown in Supplementary

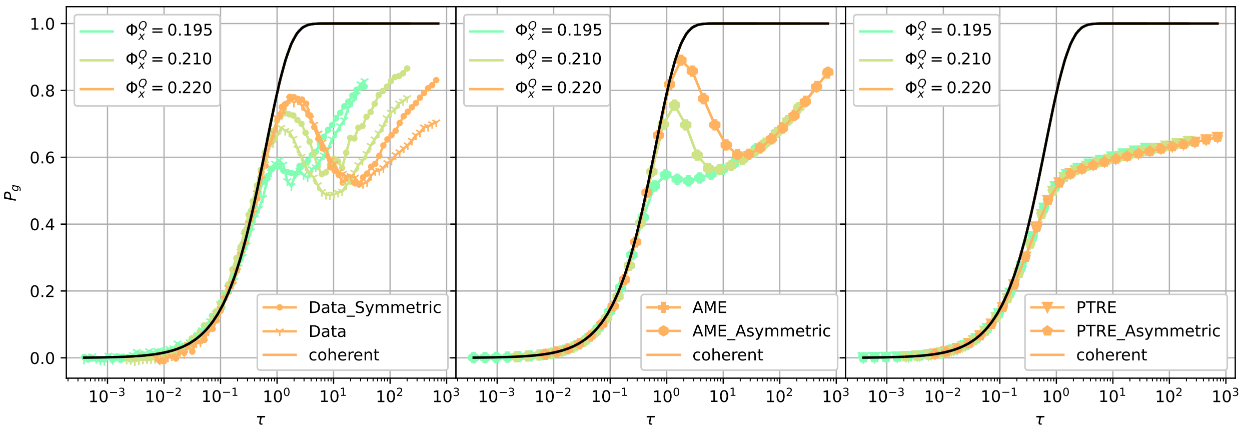

Supplementary Fig. 10. Final ground state probabilities comparing the symmetric and asymmetric sweep around Φ_z symmetry point, for experiment data on the left panel, AME simulation on the center panel and PTRE simulation on the right panel. The simulated results uses the nominal noise parameters discussed in Supplementary Note 7 and the symmetric and asymmetric sweep overlap. The experiment data presented in the main text is asymmetric in range and the simulated data in the main text is symmetric in range.

Figure 11(a), where we have denoted the actual (miscalibrated) excited state voltage as V_e (V_e'), and the corresponding inferred final ground state probability as P_e (P_e'). We found that an initial excited state population ~ 0.1 is needed to eliminate the anomalous population inversion at intermediate sweep time.

The source of imperfect ground state preparation at this particular Φ_x value is likely due to frequency collision in the coupled qubit-resonator system. In Supplementary Figure 11 (b, c), we show the bare resonator frequency f_r and the bare qubit ground to second excited state transition frequency f_{02} versus the qubit x flux bias Φ_x. It can be seen that at a certain Φ_x value close to 0.2, f_{02} crosses the resonator frequency f_r. Due to finite interaction strength between the qubit and the resonator (estimated to be about 30 MHz), the frequency collision opens another anti-crossing, which is close to the anti-crossing between the first and the second excited state of the qubit circuit. When the two anti-crossings are close, the system dynamics going through them are considerably more complex than the two-level picture, which could introduce errors in the ground state preparation. However, at other Φ_x values, when the two anti-crossings can be treated separately when they are far apart (when f_{02} is far from f_r). In this case, any population transfer to the resonator would also quickly decay to the collective ground state of the qubit-resonator system, aiding the cooling procedure.

Supplementary Note 9. SPIN BATH

Given that the Markovian master equations, AME and PTRE, failed to capture the crossover from the weak to strong coupling limit in the experiment data, it is natural to ask whether this crossover can be captured by simulating the experiment incorporating an explicit quantum environment. The spin bath is a natural choice for this quantum environment. First, much theoretical and experimental evidence points to spin impurities being the source of $1/f$ flux noise. Second, spins ferromagnetically coupled to the qubit offer an intuitive picture of the MRT phenomenon, a canonical example of the strong coupling limit. The non-zero expectation of the spins' polarization acts as an environmental bias to the qubit, resembling the reorganization energy in MRT. Fluctuations of this polarization due to the internal dynamics of the spins effectively dephase and broaden the longitudinal bias seen by the qubit, analogous to the MRT tunneling width.

A. Single spin bath

The simplest toy model for a quantum environment coupled to the system is a single spin ferromagnetically coupled to the system qubit, with an additional bath that causes thermalization of the spin. This system can be described by

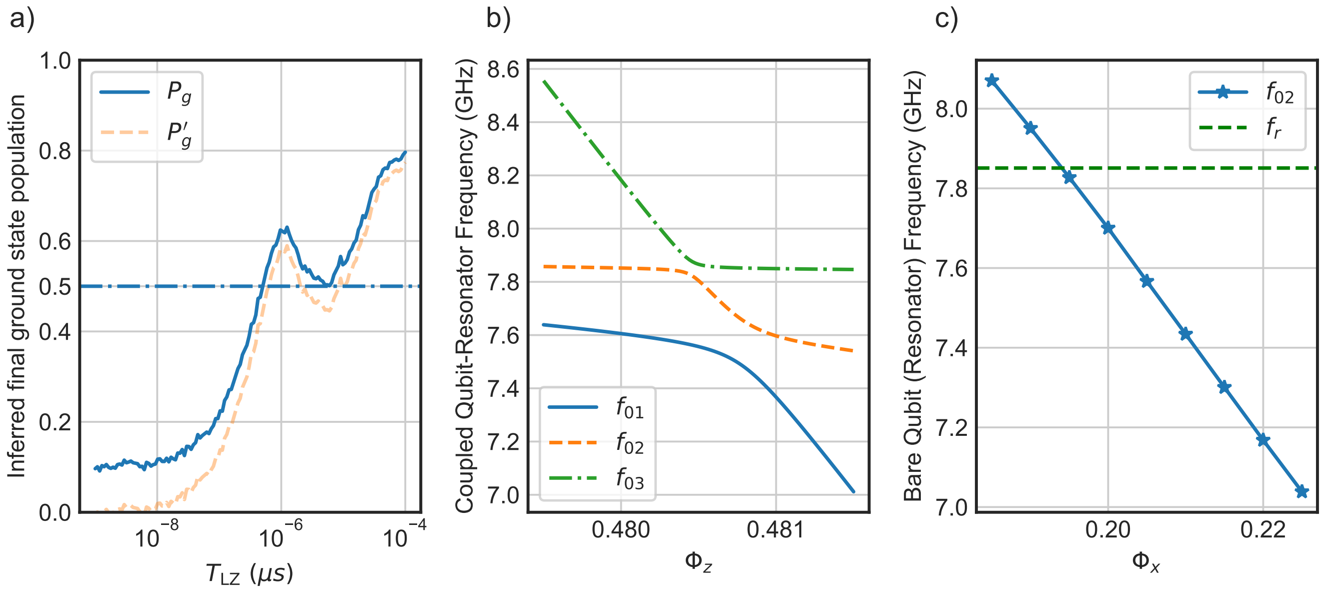

Supplementary Fig. 11. a) Inferred final ground state probabilities of the Landau-Zener measurement versus the Landau-Zener sweep time for $\Phi_x = 0.2$, P_g (blue solid line) assuming 0.1 error in ground state initialization, and P'_g (faint orange dashed line) assuming perfect ground state initialization. The data shown in the main text is P'_g. b) Numerically simulated transition frequencies of the coupled qubit-resonator system for $\Phi_x = 0.2$, near the 1-2 anti-crossing (between blue solid line and orange dashed lines) used to initialize the qubit. The additional anti-crossing between the orange dashed line and green dot-dashed lines indicates anti-crossing between the resonator and the qubit's second excited state. c) The qubit's 0-2 transition frequency versus Φ_x, with Φ_z chosen to be right at the 1-2 anti-crossing. The frequency collision between the qubit's 0-2 transition frequency and the resonator does not occur exactly around 0.2, which could be due to imperfections in the circuit modeling of the multi-level system.

the Hamiltonian

$$\begin{aligned} H &= H_q + H_{qb} + H_{qS} + H_{SB} + H_S + H_b + H_B && \text{(S12)}\\ H_{qb} &= I_p \sigma_z Q_{\Phi_z} && \text{(S13)}\\ H_{qS} &= J\sigma_z \tau_z && \text{(S14)}\\ H_S &= 0 && \text{(S15)}\\ H_{SB} &= \tau_x Q' && \text{(S16)}\\ S_{Q_{\Phi_z}}(\omega) &= S^{\rm AME}_{\Phi_z}(\omega) && \text{(S17)}\\ S_{Q'}(\omega) &= \hbar^2 \lambda \frac{1}{1+\exp\left(-\beta\hbar\omega\right)} \exp\left(-\frac{\omega}{\omega_c}\right), && \text{(S18)} \end{aligned}$$

The model Hamiltonian is understood as follows. First, the high-frequency, Markovian part of the noise is captured by the noise PSD used in the previous AME simulation. On top of this, a single spin is added to capture the effect of strong low-frequency noise. The qubit is ferromagnetically coupled to the spin with coupling strength J. The environmental spin does not have internal dynamics, but it is transversely coupled to its own environment. This environment is nearly white noise, but with the induced relaxation and excitation rate of the spin satisfying detailed balance. The strength of this noise λ is essentially the thermalization rate of the spin, in the limit where the qubit-spin coupling approaches zero. Similar to the noise PSD describing the environment of the qubit, the noise PSD for the environment of the spin also has an exponential cutoff, the role of which is essentially to ensure numerical convergence.

Next, assuming the temperature and the cutoff frequency of the spin's environment are the same as the qubit's environment, there are only two parameters to the model, the coupling strength J and the free spin thermalization rate λ. We numerically simulate the Landau-Zener experiment with the above model, initializing the system in the ground state (initialization in the thermal state does not significantly change the result). The result is shown in Supplementary Figure 12, with a range of J and λ taken in geometric steps. It can be seen that for small J and λ, the result of the single spin bath model is almost the same as the qubit AME result. This can be understood from two perspectives. First, small λ corresponds to the spin being nearly coherent and having no dynamics. Therefore its effect

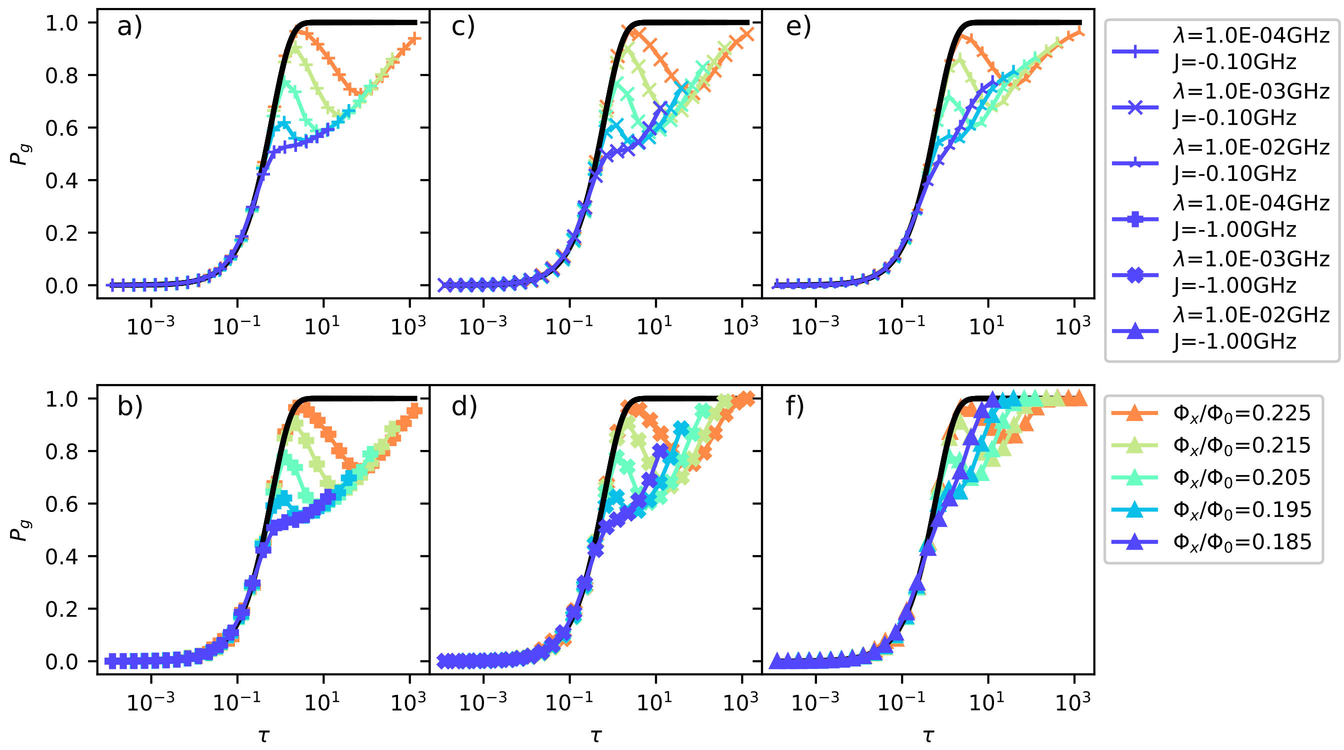

Supplementary Fig. 12. Final ground state probabilities versus the dimensionless sweep time τ, using the single spin bath model (see text). Parameters for the single spin are $\lambda = 0.0001, 0.001, 0.01$ GHz from left to right and $J/h = -0.1(1.0)$ GHz on the top(bottom).

is to merely shift the location of the anticrossing to $\epsilon + 2J = 0$. Second, for small J, the thermal equilibrium state of the spin always has its polarization $\langle \tau_z \rangle \approx 0$, irrespective of the qubit state. Therefore it has a negligible impact on the qubit. As λ and J increase, the spin bath model predicts a behavior that closely resembles the experimental data. For large Φ_x, the single spin bath model simulated ground state probably has a non-monotonic dependence versus Landau-Zener sweep time, but as Φ_x decreases, the dependence becomes monotonic and approaches the coherent limit. Among the parameters simulated, there is a good qualitative agreement between the simulated and experiment data for $\lambda = 0.001$ GHz and $J/h = 1.0$ GHz.

Further insight into the model can be obtained by looking at the instantaneous state probabilities, which are shown in Supplementary Figure 13. First, when λ is small (Supplementary Figure 13(a)), the spin is nearly coherent and simply adds an additional bias J to the qubit. This shifts the position of the anti-crossing, but does not change the final ground state probabilities, as long as J is well within the initial and final Z bias of the qubit. For small J such that $\beta|J| \ll 1$ (Supplementary Figure 13(b)), the thermal average of the spin's polarization is almost always zero, regardless of the state of the qubit, or the thermalization rate λ, Therefore the spin has a negligible effect on the qubit.

Finally, for $\beta J \gtrsim 1$, qualitative differences arise for large and small Δ (Supplementary Figure 13 (c) and (d) respectively). When Δ is large, the qubit completes the tunneling before the spin has time to re-align with the qubit. This allows thermalization to happen across the symmetrized and anti-symmetrized states of the qubit, which is why the ground state probability has a local minimum of $P_g \approx 0.5$. However, when Δ is small, the spin quickly relaxes to the opposite state as well, following the qubit. After the spin has relaxed, the qubit effectively sees a Z bias of $-2J$, which suppresses thermalization induced by the qubit's own environment. In other words, the relatively fast relaxation of the spin makes qubit tunneling irreversible. Therefore for small Δ, as the sweep time increases, the ground state probability no longer has a local minimum around 0.5.

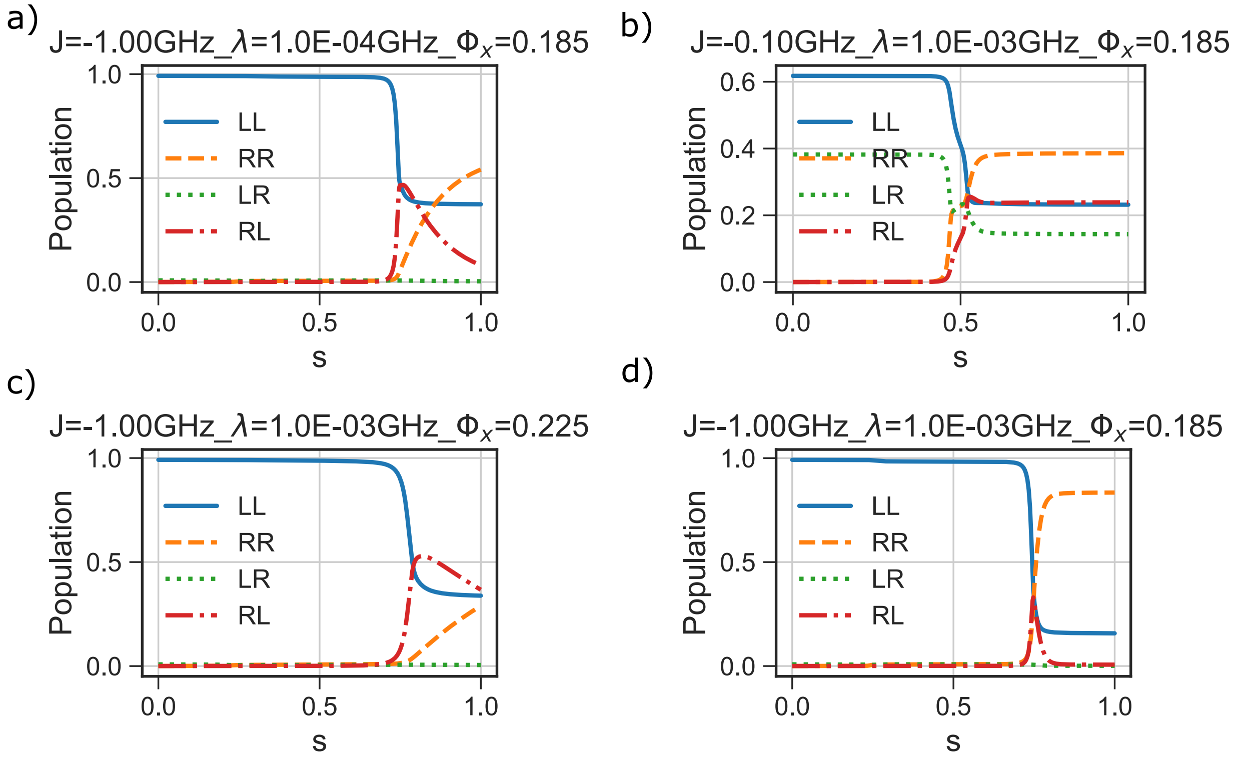

Supplementary Fig. 13. Evolution of the instantaneous qubit and spin states as a function of the normalized time $s \in [0, 1]$, for different parameters of the qubit and the spin.

B. Multiple spins

A general spin bath has a large parameter space, such as the distribution of their longitudinal and transverse fields, the distribution of couplings among them, and the distribution of couplings to their respective environments. While some of these parameters can be motivated based on plausible models of flux noise, these models often assume a macroscopic number of spins, and it is not clear whether they have an efficient numerical representation. Given this complexity, we leave it as future work to systematically explore the parameters of the spin bath and its relation to the physical $1/f$ flux noise. In this work, we adopt a simplified approach which can be seen as a quantum extension of simulating classical $1/f$ noise with two-level fluctuators [S16].

In the multi-spin model, the Hamiltonian changes from the single-spin model by making the changes given by

$$H_{qS} = \sum_{i}^{N_s} J_i \sigma_z \tau_{z,i}, \tag{S19}$$

$$H_{SB} = \sum_{i}^{N_s} \tau_{x,i} Q'_i, \text{ and} \tag{S20}$$

$$S_{Q'_i}(\omega) = \hbar^2 \lambda_i \frac{1}{1+\exp\left(-\beta\hbar\omega\right)} \exp\left(-\frac{\omega}{\omega_c}\right). \tag{S21}$$

The spin bath parameters J_i and λ_i can be chosen based on the measured $1/f$ flux noise strength. To see this, we first consider the large N_s, weak coupling limit, where the effect of the spin bath can be well captured by the noise PSD. Following Ref. [S17], the symmetrized noise PSD of the i'th spin is

$$S_i(\omega) = (1 - \langle \tau_i \rangle) \frac{2\gamma_i J_i^2}{\omega^2 + \gamma_i^2}, \tag{S22}$$

where $\langle \tau_i \rangle$ is the expectation value of spin i's longitudinal polarization, and γ_i is its thermalization rate. In general, the expectation value and the thermalization rate depend on the instantaneous qubit Hamiltonian. However, for a

small enough coupling between the qubit and the spin, the effect of the qubit on the spin is an effective longitudinal bias with strength, $-J_i\langle\sigma_z\rangle/2$. In this case, the expectation value and the thermalization rate are

$$\begin{aligned} \langle\tau_i\rangle &= \tanh\left(\beta\langle\sigma_z\rangle J_i\right), && \text{(S23)}\\ \gamma_i &= \lambda_i \exp\left(-\beta|J_i|\langle\sigma_z\rangle\right). && \text{(S24)} \end{aligned}$$

Then to obtain $1/f$ like noise with exponent α, we assume that the distribution of λ for all the spins is given by

$$\begin{aligned} P_\lambda(\lambda) &= \frac{1}{N_\lambda}\frac{1}{\lambda^\alpha}, && \text{(S25)}\\ N_\lambda &= \left(\frac{1}{-\alpha+1}\right)\left(\lambda_{\mathrm{max}}^{-\alpha+1} - \lambda_{\mathrm{min}}^{-\alpha+1}\right), && \text{(S26)} \end{aligned}$$

where $\lambda_{\mathrm{min}}, \lambda_{\mathrm{max}}$ can be chosen based on the frequency range of the noise PSD that we are interested in simulating. For simplicity, we also assume that the ferromagnetic coupling is constant for all spins, $J_i = J$. Then the collective noise PSD of the spin bath is

$$\begin{aligned} S_S(\omega) &= N_s(1-\langle\tau\rangle)\int_{\lambda_{\mathrm{min}}}^{\lambda_{\mathrm{max}}} P_\lambda(\lambda)\frac{2\gamma_i(\lambda)J^2}{\omega^2+\gamma^2(\lambda)} && \text{(S27)}\\ &= (1-\langle\tau\rangle)\frac{N_sJ^2}{N_\lambda}c^{\alpha-1}\frac{1}{\omega^\alpha}\mathcal{I} && \text{(S28)} \end{aligned}$$

where we have introduced

$$\begin{aligned} \langle\tau\rangle &= \tanh\left(\beta\langle\sigma_z\rangle J_i\right), && \text{(S29)}\\ c &= \exp(-\beta|J_i|) \text{ and} && \text{(S30)}\\ \mathcal{I} &= \int_{c\lambda_{\mathrm{min}}/\omega}^{c\lambda_{\mathrm{max}}/\omega}\frac{x^{1-\alpha}}{1+x^2}dx. && \text{(S31)} \end{aligned}$$

We can notice that for $\beta J \to 0$, the J dependence of the noise PSD primarily comes from the J^2 term. This allows us to set J based on the measured flux noise power. Comparing $S_S(\omega)$ with the symmetrized flux noise power we have

$$J = \sqrt{\frac{A^*_{\Phi_z}N_\lambda}{\mathcal{I}N_s}}I_p. \quad \text{(S32)}$$

In the above expression, the integral $\mathcal{I}$ can be evaluated at a typical ω in between λ_{max} and λ_{min}, and it is always close to 1.

To confirm the intuition that the spin bath captures the MRT parameters, we can compare the following two expressions. First, from the fluctuation-dissipation theorem and the definition of MRT width using symmetrized noise PSD in Supplementary Eq. S10, we have

$$\begin{aligned} \epsilon_p &= \frac{\beta}{2}W^2 && \text{(S33)}\\ &= \beta I_p^2 A^*_{\Phi_z}\frac{1}{-\alpha+1}\left[\left(\frac{\omega_{\mathrm{high}}}{2\pi}\right)^{-\alpha+1} - \left(\frac{\omega_{\mathrm{low}}}{2\pi}\right)^{-\alpha+1}\right]. && \text{(S34)} \end{aligned}$$

On the other hand, the effective bias applied to the qubit by the spin bath is

$$\begin{aligned} \epsilon_{SB} &= N_sJ\tanh\left(\beta J\right) && \text{(S35)}\\ &\approx N_s\beta J^2 && \text{(S36)}\\ &= \beta A^8_{\Phi_z}I_p^2\frac{1}{\mathcal{I}}\frac{1}{-\alpha+1}\left[\left(\frac{\lambda_{\mathrm{high}}}{2\pi}\right)^{-\alpha+1} - \left(\frac{\lambda_{\mathrm{low}}}{2\pi}\right)^{-\alpha+1}\right]. && \text{(S37)} \end{aligned}$$

Therefore, if we choose $\lambda_{\mathrm{max}} \gtrsim \omega_{\mathrm{max}}$ and $\lambda_{\mathrm{min}} \lesssim \omega_{\mathrm{min}}$, these two expressions, Supplementary Eq. S34 and S37 indeed match each other, up to a constant $\mathcal{I}$ that is close to unity.

As mentioned in the main text, when the low-frequency noise is not large enough, the spin bath simulation results closely resemble that of the single-qubit AME. This is exemplified in Supplementary Figure 14, where the spin bath

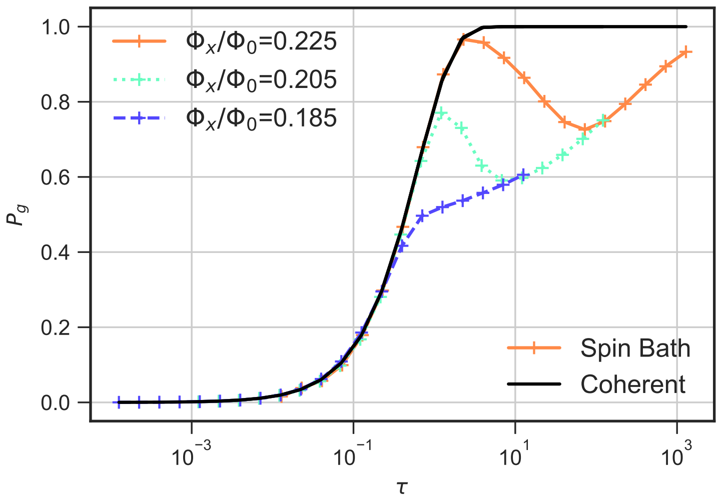

Supplementary Fig. 14. Simulated LZ final ground state probabilities versus the dimensionless sweep time τ for different Φ_x or Δ, using the spin bath model, with 3 spins and targeting the nominal $1/f$ noise amplitude.

parameters are chosen to target an $1/f^{\alpha}$ noise spectrum with the same amplitude as deduced from the decoherence measurements, therefore being 8 times smaller than the simulation presented in the main text.

[S1] Macklin, C. *et al.* A near–quantum-limited Josephson traveling-wave parametric amplifier. *Science* **350**, 307–310 (2015).

[S2] Dai, X. *et al.* Calibration of Flux Crosstalk in Large-Scale Flux-Tunable Superconducting Quantum Circuits. *PRX Quantum* **2**, 040313 (2021).

[S3] Kerman, A. J. Efficient numerical simulation of complex Josephson quantum circuits (2020). arXiv:2010.14929.

[S4] Valenzuela, S. O. *et al.* Microwave-Induced Cooling of a Superconducting Qubit. *Science* **314**, 1589–1592 (2006).

[S5] Yan, F. *et al.* The flux qubit revisited to enhance coherence and reproducibility. *Nat. Commun* **7**, 12964 (2016).

[S6] Quintana, C. M. *et al.* Observation of Classical-Quantum Crossover of $1/f$ Flux Noise and Its Paramagnetic Temperature Dependence. *Phys. Rev. Lett.* **118**, 057702 (2017).

[S7] Lanting, T. *et al.* Probing high-frequency noise with macroscopic resonant tunneling. *Phys. Rev. B* **83**, 180502 (2011).

[S8] Weber, S. J. *et al.* Coherent coupled qubits for quantum annealing. *Phys. Rev. Appl.* **8**, 014004 (2017).

[S9] Trappen, R. *et al.* Decoherence of a tunable capacitively shunted flux qubit (2023). 2307.13961.

[S10] Johansson, J. *et al.* Landau-Zener transitions in a superconducting flux qubit. *Phys. Rev. B* **80**, 012507 (2009).

[S11] Harris, R. *et al.* Experimental demonstration of a robust and scalable flux qubit. *Phys. Rev. B* **81**, 134510 (2010).

[S12] Albash, T., Boixo, S., Lidar, D. A. & Zanardi, P. Quantum adiabatic Markovian master equations. *New J. Phys.* **14**, 123016 (2012).

[S13] Javanbakht, S., Nalbach, P. & Thorwart, M. Dissipative Landau-Zener quantum dynamics with transversal and longitudinal noise. *Phys. Rev. A* **91**, 052103 (2015).

[S14] Amin, M. H. S. & Averin, D. V. Macroscopic Resonant Tunneling in the Presence of Low Frequency Noise. *Phys. Rev. Lett.* **100** (2008).

[S15] Smirnov, A. Y. & Amin, M. H. Theory of open quantum dynamics with hybrid noise. *New J. Phys.* **20**, 103037 (2018).

[S16] Dutta, P. & Horn, P. M. Low-frequency fluctuations in solids: $1/f$ noise. *Rev. Mod. Phys.* **53**, 497–516 (1981).

[S17] Shnirman, A., Schön, G., Martin, I. & Makhlin, Y. Low- and High-Frequency Noise from Coherent Two-Level Systems. *Phys. Rev. Lett.* **94**, 127002 (2005).
